# Complete genome sequences of two shipworm endosymbiont strains, *Teredinibacter turnerae* SR01903 and SR02026

**DOI:** 10.1128/mra.00265-25

**Published:** 2025-05-20

**Authors:** Mark T. Gasser, Ron Flatau, Marvin A. Altamia, Claire Marie Filone, Daniel L. Distel

**Affiliations:** 1 Johns Hopkins University Applied Physics Laboratory 70603, Laurel, Maryland, USA; 2 Ocean Genome Legacy Center, Northeastern University 1848, Boston, Massachusetts, USA; Portland State University, Portland, Oregon, USA

**Keywords:** shipworm, symbiosis, endosymbionts, intracellular bacteria

## Abstract

We present the complete genome sequences of two strains of *Teredinibacter turnerae*, SR01903 and SR02026, shipworm endosymbionts isolated from the gills of *Lyrodus pedicellatus* and *Teredo bartschi*, respectively, and derived from Oxford Nanopore sequencing. These sequences will aid in the comparative genomics of shipworm endosymbionts and symbiosis model development.

## ANNOUNCEMENT

**Fig 1:**
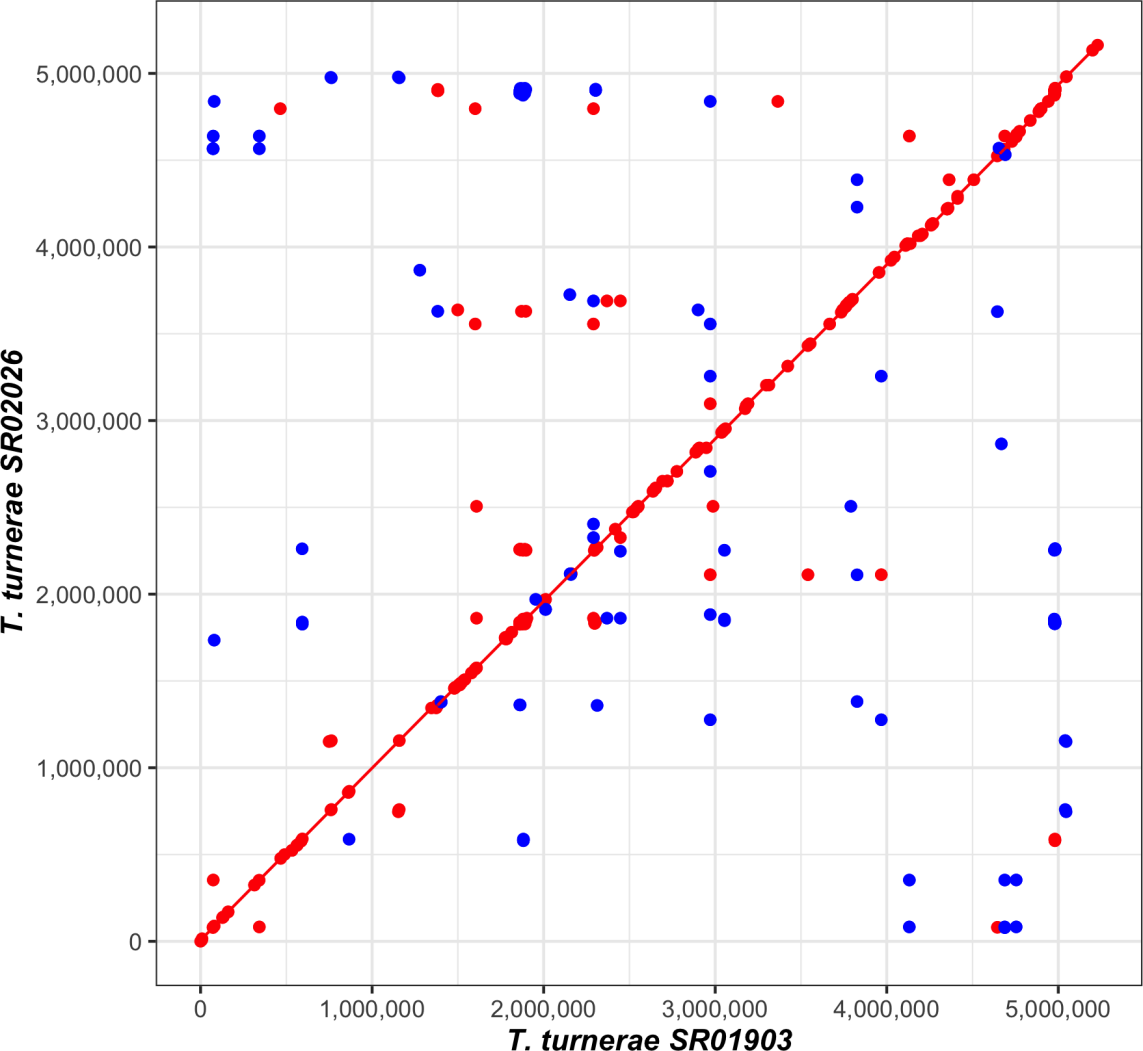
Synteny plot comparing the genome sequences of SR01903 and SR02026. A MUMmer3 plot was generated with NUCmer v3.1 (18) using NUCmer to assess synteny and completion. Minimum exact matches of 20 bp are represented as a dot with lines representing match lengths > 20 bp. Forward matches are displayed in red, while reverse matches are shown in blue.

*Teredinibacter* species (class: Gammaproteobacteria, family: Cellvibrionaceae) are cultivable intracellular endosymbionts of xylotrophic (wood-eating), bivalve wood-borers (Teredinidae) commonly called shipworms (1–4). Shipworm symbionts secrete lignocellulolytic enzymes that aid shipworm hosts in wood digestion (5, 6), have been the focus of research targeting novel enzyme and drug discovery (6–11), and are key to developing shipworm symbiotic systems as models for symbiosis research. To advance these studies, we present the complete genomes of two strains of *Teredinibacter turnerae*, the most commonly occurring shipworm symbiont species. Wood containing live specimens of *Lyrodus pedicellatus* and *Teredo bartschi* was collected from the Indian River Lagoon, Merit Island, FL (N 28.40605 W 80.66034) on 24 January 2020 and subsequently maintained in laboratory culture as in reference 12. Strain SR01903 was isolated from the gill of a single specimen of *L. pedicellatus* immediately after collection from the wild. Strain SR02026 was isolated from the gill of a fourth-generation lab-reared specimen of *T. bartschi*. Bacterial isolations were performed as in O'Connor et al*.* (5). Briefly, gills were removed by dissection and homogenized in 1.0 mL of shipworm basal medium (SBM) (13) in an autoclave sterilized glass dounce homogenizer. Homogenates were streaked onto culture plates containing 1.0% Bacto agar prepared in SBM at pH 8.0 supplemented with 0.2% w/v powdered cellulose (Sigmacell Type 101; Sigma-Aldrich) and 0.025% w/v NH_4_Cl. Plates were then incubated at 30°C. When individual colonies appeared, a single colony was picked, re-streaked, and regrown. This process was repeated until clonal isolates were achieved. Genomic DNA was extracted from the resulting clonal isolates, as in O'Connor et al*.* (5), using the Qiagen DNeasy Blood and Tissue Kit following the manufacturer’s recommended protocol for cultured cells with the exception that DNA was eluted with two 75 µL volumes of AE buffer preheated to 56°C. DNA quality and length were assessed on Tapestation (Agilent Technologies, US). Nanopore (Oxford Nanopore Technologies, UK) sequencing was performed using the Q20+ Chemistry Ligation Sequencing Kit (SQK-LSK112) and sequenced on a MinION (Mk1B) instrument with an R10.4 (FLO-MIN112) flow cell. Bases were called using Guppy v6.4.6 with the high-accuracy algorithm and the default read quality filtering. Adapters were trimmed from reads using Porechop v0.2.4 (https://github.com/rrwick/Porechop) and filtered to remove reads less than 1 Kb using Filtlong v0.2.1 (https://github.com/rrwick/Filtlong). *De novo* assembly was performed with Flye v2.9.2 (https://github.com/fenderglass/Flye) (14), followed by contig correction and consensus generation with Racon v1.5.0 (https://github.com/lbcb-sci/racon) and Medaka v1.8.0 (https://github.com/nanoporetech/medaka). Assemblies were then circularized and rotated to start at *dnaA* predicted by prodigal v2.6.3 (15) with Circlator v1.5.5 (https://github.com/sanger-pathogens/circlator) (16). All software were run using default settings unless otherwise noted. Chromosomal assemblies were produced for both strains and annotated using the NCBI Prokaryotic Genome Annotation Pipeline (Table 1) (17). The primary sequences were 98.67% identical based on the calculated average nucleotide identity (18) and highly syntenic (Fig. 1) (19). Both strains are greater than 98% identical to *T. turnerae T7901* (1), confirming their taxonomic identity.

**TABLE 1:** Genome sequencing, assembly, and annotation of Teredinibacter strains

Strain	SR01903	SR02026
Host	*L. pedicellatus*	*T. bartschi*
Reads	77,041	253,198
Bases (M)	289.9	306.2
Read N50	11,088	2,331
Assembly (bp)	5,229,132	5,163,139
Coverage	52×	44×
GC content (%)	50.9	50.9
Genes	4,207	4,142
CDS	4,152	4,086
rRNA (complete)	3	3

## Data Availability

The complete genome sequences for SR01903 and SR02026 have been deposited in GenBank under the accession numbers CP149818 and CP149819, respectively. The Oxford Nanopore sequencing reads are available from the NCBI Sequence Read Archive (SRA) under the accession numbers SRR28421271 and SRR28421270, respectively.
